# Autonomic and physiological stress responses in navy divers: the protective role of diving experience

**DOI:** 10.3389/fphys.2025.1642779

**Published:** 2025-08-18

**Authors:** Yu-Ju Chen, Yuan-Sheng Tzeng, Shih-En Tang, Chi-Rong Li, Shu-Yu Wu, Kun-Lun Huang

**Affiliations:** ^1^ National Defense Medical Center, Taipei, Taiwan; ^2^ Kaohsiung Armed Forces General Hospital Zuoying Branch, Kaohsiung, Taiwan; ^3^ Tri-Service General Hospital Department of Medicine, Taipei City, Taiwan; ^4^ Taichung Hospital, Ministry of Health and Welfare, Taichung, Taiwan

**Keywords:** autonomic nervous system, heart rate variability, salivary cortisol, amylase activity, psychological distress, deep diving

## Abstract

**Introduction:**

Deep diving presents significant physiological stress, yet reliable indicators for monitoring autonomic and stress responses remain underdeveloped. This study examined how prior deep diving experience influences autonomic regulation and stress biomarkers during a simulated dive to a depth of 220 feet.

**Methods:**

Twenty-eight Navy divers (15 experienced, 13 novice) underwent psychological assessments (perceived stress, anxiety, fear) and salivary biomarker analysis (cortisol, amylase) pre- and post-dive. Heart rate variability (HRV), including linear (rMSSD, HF) and non-linear indices (SD1, SD1/SD2, sample entropy), was measured at four dive stages (pre-dive, bottom, decompression, and post-dive).

**Results:**

After adjusting for age and perceived stress, experienced divers exhibited greater post-dive reductions in cortisol (*p* = 0.015) and amylase (*p* = 0.022). Additionally, after controlling for age, perceived stress, and respiratory rate, they also demonstrated significantly higher parasympathetic activity (*p* ≤ 0.001) and greater HRV complexity (sample entropy, *p* = 0.023) during decompression. No significant differences were found in self-reported psychological stress.

**Discussion:**

Diving experience facilitates enhanced autonomic control and stress adaptation. These findings support the use of real-time physiological monitoring and experience-based training protocols to mitigate risk during deep dives.

## Introduction

Naval deep-diving, essential for underwater military operations and search-and-rescue missions, exposes divers to high-pressure environments that require excellent physical and psychological robustness. These diving activities pose inherent risks such as barotrauma, high-pressure nervous syndrome, nitrogen narcosis, oxygen toxicity, and decompression sickness, all significant concerns for diving safety (Bove, 2014). While training is recognized as crucial for enhancing deep diving skills (Lemaître et al., 2008), a clear understanding of psychophysiological indicators for monitoring training effectiveness remains a gap.

Submersion affects the human body profoundly; water buoyancy causes a fluid shift that increases central blood volume, enhancing venous return, stroke volume, and cardiac output. This fluid redistribution, coupled with hydrostatic pressure, triggers a diving reflex characterized by increased parasympathetic and decreased sympathetic activity, leading to a slower heart rate (Costalat et al., 2017; Hagen, 2018; Panneton and Gan, 2020). These physiological responses are crucial adaptive mechanisms for coping with underwater environments (Moore et al., 1972; Park et al., 1999; Beatty et al., 2024). Beyond the intense physiological demands, psychological resilience is crucial for divers. Research consistently indicates that experienced divers exhibit superior emotional regulation due to extensive training, critical for psychological preparedness and effective coping with the substantial psychophysiological demands encountered in deep-water environments (Bonnet et al., 2008). This highlights the vital role of comprehensive training programs that not only support physical conditioning and mental health strategies but also account for individual psychological traits such as risk-seeking tendencies, which may influence diver behavior and performance under pressure (Niewiedział et al., 2018).

The intense physical and psychological stress experienced during deep dives activates the hypothalamic-pituitary-adrenal (HPA) and sympathetic-adrenal-medullary (SAM) axes, elevating stress-indicative hormones like cortisol and norepinephrine (Anegg et al., 2002). Recent research suggests that measuring salivary cortisol and alpha-amylase provides a practical method for assessing stress in offshore saturation diving (Monnoyer et al., 2021). This body of work demonstrates the profound effects of high-pressure environments on the endocrine system, suggesting that hormonal imbalances may stem from psychological and emotional responses, especially among novices undergoing initial training (Vaernes et al., 1982; Vaernes and Darragh, 1982).

Heart rate variability (HRV) serves as a reliable indicator of autonomic nervous system (ANS) functioning in divers, reflecting physiological stress responses and adaptation to underwater pressures (Kurita et al., 2002; Malinowski et al., 2023). Adjustments in blood distribution and autonomic responses significantly influence cardiac regulation and are evident in HRV indices like high frequency (HF) and root mean square of successive differences (rMSSD) (Park et al., 1999; Schipke and Pelzer, 2001; Berry et al., 2017; Sato et al., 2017). Notably, deeper dives enhance sympathetic activity, impacting HRV and underlining the need for precise autonomic monitoring (Lund et al., 2000). Moreover, the psychological stress associated with diving can further excite sympathetic nerve activity, impacting HRV (Schipke and Pelzer, 2001).

Linear HRV indices are more commonly used in diving research due to their established application and ease of interpretation. However, to better assess the dynamic responses of the autonomic nervous system (ANS) to the stresses of diving while minimizing the influence of breathing patterns on heart rate, the use of non-linear HRV indices is also advocated (Lundell and Ojanen, 2023). Non-linear HRV indices, such as SD1 and the SD1/SD2 ratio derived from the Poincaré plot, offer valuable insights into autonomic regulation, with SD1 reflecting short-term heart rate variability and the SD1/SD2 ratio capturing the balance between short- and long-term dynamics. Research in diving contexts has shown that SD1 and high frequency (HF) components increase concurrently, indicating enhanced parasympathetic nervous system activity under hyperbaric conditions (Shaffer and Ginsberg, 2017). Moreover, Approximate Entropy (ApEn), which assesses the complexity and regularity of heart rate time-series, reflecting adaptability, has been observed to increase at depths of 30 m during ascent and post-dive in experienced divers using closed-circuit rebreathers (Lipsitz, 2002; Lafère et al., 2021; Lundell and Ojanen, 2023). Conversely, a study measuring ApEn at various depths (33–200 ft) using SCUBA gear reported no significant changes from baseline, except for a decrease at 99 ft (Noh et al., 2018), highlighting inconsistent results. Prior findings suggest that Sample Entropy (SampEn), known for its lower susceptibility to biases and higher reliability in smaller samples, may offer more accurate insights into cardiac complexity than ApEn (Richman and Moorman, 2000). Considering the typical limitations in sample size and the need for repeated time-series measurements in diving research, SampEn is likely more suitable for effectively assessing physiological responses among divers.

Simulated deep diving training is essential for preparing naval personnel to perform effectively in deep-water operations and submarine missions. Despite its importance, such training can challenge ANS stability and induce psychological stress, with the role of cardiac autonomic regulation in diving efficacy and decompression sickness prevention still unclear. This study examines how prior deep diving experience affects autonomic regulation and stress biomarkers during simulated dives to 220 feet, aiming to inform and optimize training protocols and improve safety and operational outcomes in naval diving contexts.

## Methods and materials

This prospective, repeated-measures design study was conducted at the military simulated diving center in southern Taiwan and approved by the institutional review board (TSGHIRB-A202105106).

### Participants

We recruited Navy divers scheduled to undergo a 220-foot simulated deep dive using a helium-oxygen gas mixture in a wet hyperbaric chamber from a simulated diving training center. All participants were healthy, normotensive, met military fitness standards, and free from medication use. Participant characteristics are detailed in Table 1. The primary investigator obtained informed consent from each participant prior to data collection.

**TABLE 1:** Demographics of participants/simulated divers.

Variables	All (*n* = 28)	Experienced (*n* =15)	Novice (*n* =13)	*p*
	Mean (SD)/Median (IQR)/n (%)			
Age (year)	28.8 (5.9)	32.5 (5.2)	24.5 (3.1)	<0.001^a^
Gender				
Male	27 (96.9)	15 (100)	12 (92.3)	0.464^c^
Female	1 (3.6)	0 (0)	1 (13)	
BMI(kg/m^2^)	24.1 (2.3)	24.37 (2.36)	23.83 (2.35)	0.550^a^
Smoking				
Yes	10 (39.3)	3 (20)	7 (53.8)	0.114^c^
No	18 (60.7)	12 (80)	6 (46.2)	
Alcohol consumption				
Yes	6 (67.8)	5 (33.3)	1 (7.7)	0.173^c^
No	22 (32.1)	10 (66.7)	12 (92.3)	
Diving experience (year)	8.0 (3.3–10.0)	10.0 (9.0–12.0)	4.0 (2.5–4.5)	<0.001^b^
Weekly exercise (mins)	300 (157–600)	300 (150–540)	525 (202–615)	0.254^b^

Notes: SD, standard deviation; IQR, interquartile range.

^a^ = independent t-test.

^b^ = Mann-Whitney U test.

^c^ = Chi-square test.

### Study protocol

**FIGURE 1:**
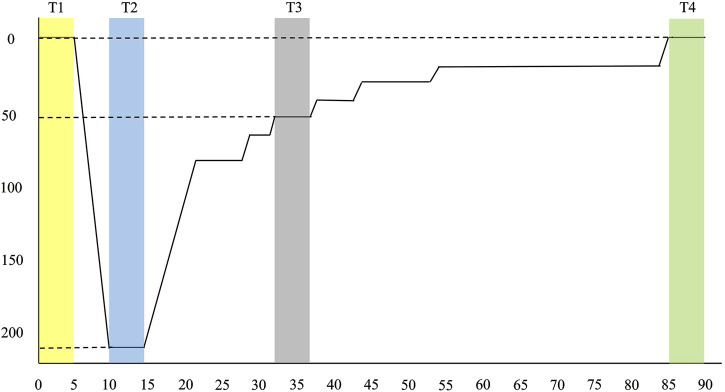
Diving profile. Modified from U.S. Navy Diving Manual, Revision 7 (2016). Divers descended to a maximum depth of 220 feet with a bottom time of 5 min. Including ascent and decompression stops, the total dive duration was 90 min. T1 to T4 represents four designated time points for 5-min HRV recordings: T1 (pre-dive), T2 (bottom), T3 (decompression), and T4 (post-dive).

All simulated dives in this study followed a standardized 220-foot diving profile, adapted from the modified decompression table of the *U.S. Navy Diving Manual, Revision 7* (U.S.DepartmentoftheNavy, 2016). The whole sequence, including pre-entry, chamber entry, descent, bottom time, ascent (decompression), and surfacing, lasted a total of 90 min (see Figure 1). Breathing gas mixtures were administered according to depth and stage to optimize safety and physiological performance. During descent, divers initially breathed compressed air until reaching approximately 50 feet. From that point through the bottom phase at 220 feet, the training officer provided divers an 83% helium and 17% oxygen (He/O_2_) mixture to mitigate nitrogen narcosis and reduce breathing resistance under high pressure. During the decompression ascent, the training officer switched the mix to 50% nitrogen and 50% oxygen (N_2_/O_2_) to facilitate inert gas elimination while minimizing the risk of oxygen toxicity. In the final decompression stages at shallower depths, divers received 100% oxygen to enhance nitrogen off-gassing and accelerate decompression safely. Participants conducted all dives before noon, with hyperbaric chamber temperatures between 22°C and 25°C to ensure thermal stability during depth transitions. No cases of decompression sickness or other adverse events occurred during or after the simulated dives, which were conducted under standardized and medically supervised conditions.

With informed consent, all participants completed a demographic questionnaire, including age, gender, height, weight, smoking and alcohol consumption habits, diving experience, and weekly exercise duration. The investigator conducted baseline assessments before the dive while participants sat and rested. These included self-reported psychological measures (perceived stress, anxiety, and fear), collection of saliva samples for stress biomarkers (cortisol and amylase activity), and recordings of HRV. Post-dive, the investigator repeatedly conducted psychological assessments (anxiety and fear) and a saliva sample collection. HRV was continuously monitored throughout the dive using a wearable Polar V800 device.

## Measurements

### Psychological measures (perceived stress, anxiety, fear)

The investigator used the 10-item Perceived Stress Scale (PSS-10) to assess Navy divers’ perceived stress levels before diving. Developed by Cohen and Williamson (Cohen, 1988), the PSS-10 measures individuals’ subjective appraisal of stress and their perceived ability to cope with environmental demands. Each item is rated on a five-point Likert scale, ranging from 0 (“Never”) to 4 (“Always”), with total scores ranging from 0 to 40. Higher scores indicate greater perceived stress. The PSS-10 has demonstrated strong reliability and validity in nonclinical populations (Roberti et al., 2006), and internal consistency in this study was high (Cronbach’s α = 0.84).

Two visual analog scales (VAS), widely recognized for their simplicity, ease of administration, and reliability in assessing psychological distress in occupational settings (Dutheil et al., 2024), were used to evaluate participants’ self-reported levels of anxiety and fear before and after the dive. Each scale consisted of a 10-cm horizontal line, anchored with the prompt, “How anxious/fearful do you feel right now?” The left end of the line, marked as 0, was labeled “Not at all,” indicating no anxiety or fear, and the right end, marked as 100, was labeled “Extremely anxious/fearful,” indicating a very intense emotional state.

### Salivary cortisol and amylase activity

Saliva samples were collected in the morning using Salivette tubes (Sarstedt, Cat. #51.1534). Participants were instructed to avoid eating, drinking (except water), brushing their teeth, or using mouthwash for at least 60 min before sample collection and to ensure that at least 2 h had passed since breakfast to minimize circadian influences and variability in saliva composition. For the collection, participants placed the sterile cotton swab from the Salivette tube into their mouth and gently chewed it for 1–2 min to stimulate saliva flow. The swab was then returned to the tube, sealed, and immediately stored on dry ice before transport to the laboratory.

Before testing, samples were thawed to room temperature, centrifuged at 1,500 × g for 2 min at 4°C, and the investigator transferred the retrieved saliva to 1.5 mL tubes and stored at −80°C until analysis. Salivary cortisol levels were determined using an ELISA kit (Cayman Chemical, United States), with samples diluted 1:10 using the provided diluent. Amylase activity was measured using a kinetic colorimetric assay (Novus Biologicals LLC, Bio-Techne, United States), with saliva samples diluted 1:20 in double-distilled water. All assays were conducted following the manufacturers’ protocols.

### Heart rate variability, HRV

HRV data were recorded using the Polar V800 system with a sensor integrated into a specially designed Polar diving suit (Polar Electro, Oulu, Finland). This system has been validated for accurately capturing RR intervals and HRV parameters compared to electrocardiograms (ECGs) under controlled conditions (Giles et al., 2016). During the dive, participants remained seated and as still as possible to minimize motion artifacts. HRV data were continuously recorded beginning 10 min before chamber entry and continuing through all dive stages, using a 1,000 Hz sampling rate. From this continuous dataset, the investigator extracted four 5-min HRV segments, following the standards of HRV measurement outlined by the Task Force of the European Society of Cardiology and the North American Society of Pacing and Electrophysiology (Malik, 1996), to represent key stages: pre-dive (5 min before descent), bottom (5 min at maximum depth), decompression (5-min stop at 50 feet during ascent), and post-dive (5 min after surfacing). Data were analyzed using Kubios HRV software version 3.4 (University of Eastern Finland, Kuopio, Finland) to obtain time-domain, frequency-domain, and non-linear HRV indices.

Time-domain linear HRV indices comprise mean heart rate (MHR), the standard deviation of normal-to-normal intervals (SDNN), and the root mean square of successive differences between adjacent NN intervals (rMSSD). Frequency-domain HRV analysis was conducted using Fast Fourier Transform (FFT) to quantify the power of spectral components: low-frequency (LF) power ranging from 0.04 to 0.15 Hz, and high frequency (HF) power ranging from 0.15 to 0.40 Hz. Non-linear HRV indices, calculated from the Poincaré plot, included SD1 and the SD1/SD2 ratio. SD1 represents the dispersion of points perpendicular to the line of identity, reflecting short-term heart rate variability and parasympathetic activity. SD2 (standard deviation 2) means the dispersion along the identity line, corresponding to long-term variability. The SD1/SD2 ratio provides an index of the balance between short-term and long-term autonomic dynamics.

Controlling the respiratory rate is recommended for accurate HRV analysis (Grossman and Taylor, 2007). Due to equipment limitations, the respiratory rate during all diving stages was estimated from RR intervals using validated algorithms in Kubios HRV software (Rogers et al., 2022) and statistically controlled for in the HRV analyses.

### Statistical analysis

Demographics and pre-dive psychophysiological data were summarized using descriptive statistics, including frequency, percentage, mean, standard deviation, median, and the range of quantile interval (IQR). We assessed data normality using the one-sample Kolmogorov-Smirnov test. Independent t-tests were used for normally distributed variables, while Mann-Whitney U tests were applied for non-normally distributed variables to compare experienced and novice divers. Skewed HRV indices, specifically HF and LF (ranging from 2.00 to 3.02), were normalized using natural logarithm (ln) transformation.

Analyses were performed using Generalized Estimating Equations (GEE) in SPSS (Version 19.0; SPSS Inc., Chicago, IL, United States), applying an identity link function to model relationships between independent and dependent variables over repeated measures. The effects of group (deep diving experience: experienced vs. novice) and Group by Time (experience x dive stage) interactions on psychophysiological outcomes were evaluated. Confounding factors, including age, pre-dive perceived stress, and respiratory rate, were controlled to ensure accurate assessment of physiological responses across dive stages and between diver groups (Malik, 1996; Grossman and Taylor, 2007).

An independent correlation structure was selected for the GEE analyses due to low or inconsistent within-subject correlations, similar QIC values across alternative structures, and the small sample size, favoring model simplicity. Results are reported as beta coefficients (*β*) with 95% confidence intervals, with significance set at *p* < 0.05.

## Results

### Demographics of participating divers

We recruited 28 Navy divers from a military simulated diving center in southern Taiwan, comprising 27 male and one female divers (Table 1). The participants had an average age of 28.8 ± 5.9 years and a BMI of 24.1 ± 2.3. Among them, 39.3% were current smokers and reported a median weekly exercise time of 383.8 ± 253.3 min. The median diving experience was 8.0 years (IQR: 3.3–10.0) years of diving experience. Of the participants, 15 were classified as experienced deep divers, having dived to depths greater than 220 feet, and 13 were novice deep divers, undertaking their first deep dive. Significant differences were observed between the groups in age (32.5 ± 5.3 vs. 24.5 ± 3.1 years, *p* < 0.001) and diving experience (10.0 [9.0–12.0] vs. 4.0 [2.5–4.5] years, *p* < 0.001). No significant differences were found in BMI, exercise time, or other lifestyle habits (*p* > 0.05).

### Comparison of pre-diving psychophysiological states: experienced versus novice divers

At the pre-dive stage, significant psychophysiological differences were observed between experienced and novice divers (Table 2), with cortisol levels significantly higher in experienced divers (164.05 ± 92.41 ng/mL) than in novices (95.13 ± 65.45 ng/mL, *p* = 0.034). Conversely, amylase activity was lower in experienced divers (1.08 [1.00–1.19] U/mL) than in novices (1.23 [1.06–1.28] U/mL, *p* = 0.034). No significant differences were found between groups of perceived stress, anxiety, fear, and HRV indices (all *p* > 0.05).

**TABLE 2:** Comparison the psychophysiological status between experienced or novice divers at the pre-diving stage.

Variables	All (*n* = 28)	Experienced (*n* = 15)	Novice (*n* = 13)	*p*
	Mean (SD)/Median (IQR)			
PSS	10.50 (5.35)	9.3 (5.7)	11.8 (4.7)	0.222^a^
Anxiety (VAS 1–10)	0.15 (0.00–1.78)	0.00 (0.00–1.80)	0.2 (0.00–1.85)	0.339^b^
Fear (VAS 1–10)	0.15 (0.00–0.88)	0.00 (0.00–0.50)	0.2 (0.00–1.35)	0.339^b^
Cortisol (pg/mL)	132.05 (86.93)	164.05 (92.41)	95.13 (65.45)	0.034^a^
Amylase activity (U/mL)	1.13 (1.02–1.24)	1.08 (1.00–1.19)	1.23 (1.06–1.28)	0.033^b^
MHR	87.4 (16.1)	92.31 (19.58)	81.67 (8.43)	0.081^a^
SDNN (ms)	36.8 (14.6)	32.74 (17.84)	41.44 (7.93)	0.117^a^
rMSSD (ms)	29.8 (18.2)	25.17 (21.83)	35.22 (11.26)	0.147^a^
LF ln (ms^2^)	6.56 (0.88)	6.31 (1.01)	6.86 (0.61)	0.097^a^
HF ln (ms^2^)	5.37 (1.20)	4.88 (1.33)	5.93 (0.75)	0.019^a^
SD1	21.13 (12.86)	17.83 (15.47)	24.94 (7.98)	0.148^a^
SD1/SD2	0.42 (0.14)	0.37 (0.14)	0.48 (0.14)	0.059^a^
SampEn	1.48 (0.37)	1.38 (0.39)	1.60 (0.33)	0.125^a^

Notes: SD, standard deviation; IQR, range of quantile interval; PSS, perceived stress scale; VAS, visual analog scale; MHR, mean heart rate; SDNN, standard deviation of normal-to-normal intervals, rMSSD, the root mean square of successive differences; LF, low frequency of heart rate variability; HF, high frequency of heart rate variability, ln, natural logarithm, SD1, Standard deviation 1 is the standard deviation of the distances of each data point from the line of identity (y = x) in the Poincaré plot; SD2, Standard Deviation 2 is standard deviation along the line of identity in the Poincaré plot; SD1/SD2, ratio, the ratio of short to long-term variability; SampEn, Sample entropy.

^a^ = independent t-test.

^b^ = Mann-Whitney U test.

### Examining differences in pre- and post-dive psychological and physiological stress responses between experienced divers and novices

GEE analysis, controlling for age and pre-dive perceived stress, evaluated the effect of deep diving and experience on psychophysiological measures (Table 3). As no significant interaction effects were found for anxiety or fear (all *p* > 0.05), only main effects were presented in Table 3. Self-reported anxiety and fear also remained unchanged post-dive (*p* = 0.242 and 0.211, respectively).

**TABLE 3:** Psychophysiological changes pre- and post-dive in novice vs. experienced divers.

Variables	Anxiety				Fear				Cortisol				Amylase			
	*β*	95% CI		*p*	*β*	95% C*I*		*p*	*β*	95% CI		*p*	*β*	95% CI		*p*
Age	0.003	−0.08	0.08	0.937	0.02	−0.03	0.07	0.437	2.84	−1.82	7.49	0.233	0.003	−0.01	−0.01	0.596
PSS	0.09	0.04	0.15	0.001	0.10	0.04	0.15	0.001	0.01	−3.21	3.22	0.997	−0.01	−0.02	0.01	0.249
Post- vs. pre-diving	−0.38	−1.22	0.08	0.242	−0.33	−0.84	0.19	0.211	−0.36	−26.98	26.25	0.979	−0.21	−0.17	0.03	0.703
Experienced vs. novices	0.15	−0.66	0.77	0.748	−0.11	−0.58	0.36	0.648	46.64	−17.29	110.20	0.153	−0.03	−0.42	−0.001	0.049
Experienced x stage									−63.27	−114.03	−12.51	0.015	−0.29	−0.54	−0.04	0.022

Notes: The interaction effects for anxiety and fear were non-significant (p > 0.05) and thus excluded; only the main effects are presented. PSS, perceived stress scale.

After adjusting for age and pre-dive perceived stress, no significant overall changes in cortisol or amylase levels were observed post-dive (*p* > 0.05). However, experienced divers showed significantly lower amylase levels than novices (*β* = −0.03, *p* = 0.049). Additionally, significant interactions between diver group (experienced vs. novice) and dive stage (pre-dive vs. post-dive) were found for both cortisol (*β* = −63.27, *p* = 0.015) and amylase activity (*β* = −0.29, *p* = 0.022), indicating that changes in these biomarkers from pre- to post-dive differed by diving experience.

### Group by time effects on linear and non-linear HRV indices across deep dives: experienced versus novice divers

**FIGURE 2:**
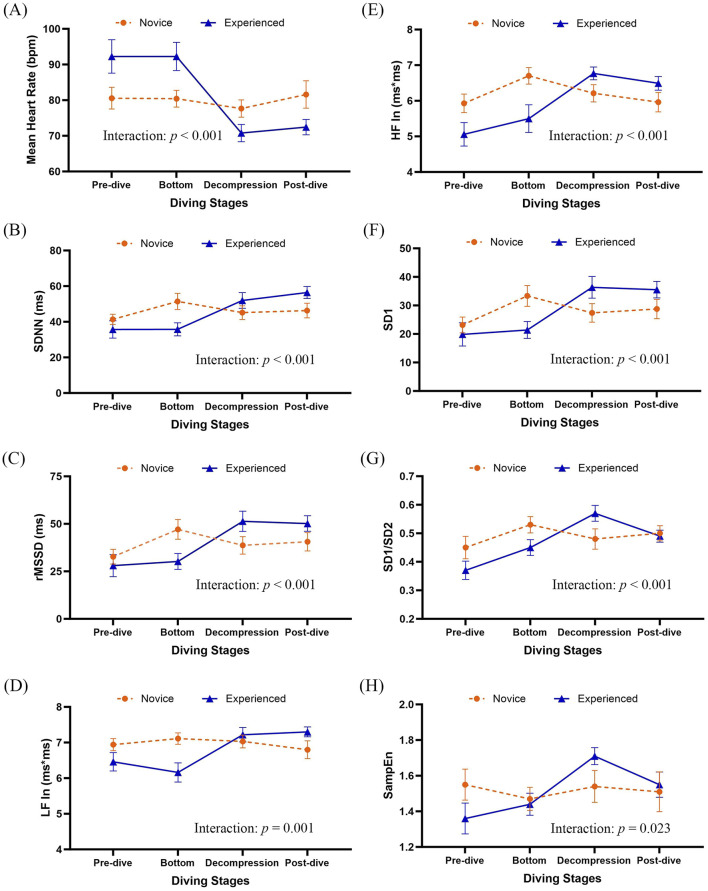
Group means with 95% confidence intervals for HRV indices across diving stages. Results were derived from generalized estimating equation (GEE) models adjusted by age, pre-dive perceived stress (PSS), and respiratory rate. Group-by-diving stages interactions were significant for several HRV parameters (see Table 4). **(A)** Mean heart rate, **(B)** SDNN, **(C)** rMSSD, **(D)** natural log-transformed LF power (LF In), **(E)** natural log-transformed HF power (HF In), **(F)** SD1, **(G)** SD1/SD2, and **(H)** SampEn. SDNN, standard deviation of normal-to-normal intervals; rMSSD, the root mean square of successive differences; LF, low frequency of heart rate variability; HF, high frequency of heart rate variability; ln, natural logarithm; SD1, Standard deviation 1 is the standard deviation of the distances of each data point from the line of identity (y = x) in the Poincaré plot; SD2, Standard Deviation 2 is standard deviation along the line of identity in the Poincaré plot; SD1/SD2 ratio, the ratio of short to long-term variability; SampEn, Sample entropy.

After controlling age, baseline perceived stress, and respiratory rate, GEE analyses examined the effects of diving experience, diving stages, and their interactions on linear HRV indices (Table 4; Figure 2). Significant stage effects were observed across all linear HRV indices (*p* < 0.05), with SDNN (*β* = 10.09, *p* = 0.033), rMSSD (*β* = 14.38, *p* = 0.006), and HF ln (*β* = 0.77, *p* = 0.016) showing notable increases at the bottom stage. A significant main effect of group was also found for mean heart rate (MHR), with experienced divers exhibiting a higher MHR than novices (*β* = 11.70, *p* = 0.045). Significant interaction effects between diver experience and diving stages were found for all linear HRV indices (*p* ≤ 0.001; Figures 2A–F). Experienced divers displayed more significant reductions in MHR from pre-dive to the decompression and post-dive stages compared to novices (*β* = −18.50, *p* < 0.001 and *β* = −20.20, *p* = 0.001, respectively). Additionally, rMSSD increased more significantly from pre-dive to the decompression stage in experienced divers compared to novices (*β* = 17.34, *p* = 0.022). Both LF ln and HF ln indices showed significant interaction effects across diving stages (*p* = 0.001 and *p* < 0.001, respectively; Figures 2D,E), with LF ln increasing post-dive (*β* = 0.98, *p* = 0.018). HF ln increased during decompression (*β* = 1.40, *p* = 0.004) and post-dive (*β* = 1.43, *p* = 0.009) in experienced divers compared to novices.

**TABLE 4:** GEE Analysis of group x time effects on linear RV indices across simulated diving stages in experienced vs. novice divers.

Variables	MHR				SDNN				rMSSD				LF ln				HF ln			
	*β*	95% CI		*p*	*β*	95% CI		*p*	*β*	95% CI		*p*	*β*	95% CI		*p*	*β*	95% CI		*p*
Age	0.14	−0.41	0.69	0.611	−0.76	−1.43	−0.09	0.027	−0.85	−1.71	0.01	0.053	−0.03	−0.06	−0.01	0.018	−0.05	−0.10	−0.01	0.022
PSS	0.49	−0.20	1.19	0.165	−0.07	−0.67	0.52	0.808	−0.27	−1.11	0.58	0.534	0.01	−0.02	0.03	0.686	−0.02	−0.07	0.02	0.227
RR	0.59	−0.48	1.66	0.282	−1.73	−2.76	−0.70	0.001	−0.44	−1.74	0.87	0.514	−0.13	−0.19	−0.07	<0.001	−0.11	−0.20	−0.02	0.019
Diving stage				<0.001				0.001				<0.001				0.014				<0.001
4 vs. 1	1.03	−7.71	9.76	0.818	4.94	−5.16	15.05	0.338	7.89	−4.71	20.49	0.220	−0.14	−0.69	0.41	0.615	0.03	−0.71	0.77	0.934
3 vs. 1	−2.91	−6.42	0.60	0.104	3.80	−3.63	11.23	0.316	5.99	−2.65	14.63	0.174	0.09	−0.41	0.59	0.724	0.28	−0.35	0.91	0.385
2 vs. 1	−0.15	−3.97	3.67	0.937	10.09	0.83	19.35	0.033	14.38	4.04	24.71	0.006	0.18	−0.30	0.66	0.470	0.77	0.14	1.39	0.016
Experienced vs. novices	11.70	0.25	23.15	0.045	−5.64	−14.81	4.60	0.373	−4.70	−20.14	10.73	0.550	−0.48	−1.10	0.15	0.135	−0.87	−1.78	0.03	0.059
Experienced x diving stage				<0.001				<0.001				<0.001				0.001				<0.001
Experienced vs. novices at Stage 4	−20.85	−33.17	−8.52	0.001	15.77	−0.25	31.79	0.054	14.28	−4.84	33.40	0.143	0.98	0.17	1.79	0.018	1.40	0.35	2.44	0.009
Experienced vs. novices at Stage 3	−18.57	−26.28	−10.86	<0.001	12.44	−0.98	25.86	0.069	17.34	2.54	32.15	0.022	0.67	−0.10	1.43	0.088	1.43	0.46	2.39	0.004
Experienced vs. novices at Stage 2	−1.83	−10.72	7.05	0.590	−10.07	−24.43	4.29	0.169	−12.17	−27.85	3.50	0.128	−0.48	−1.41	0.45	0.312	−0.33	−1.40	0.73	0.543

Notes: MHR, mean heart rate; SDNN, standard deviation of normal-to-normal intervals; rMSSD, the root mean square of successive differences; LF, low frequency of heart rate variability; HF, high frequency of heart rate variability; ln, natural logarithm; PSS, perceived stress scale; RR, respiratory rate; Diving Stage 1, pre-dive; Stage 2, bottom; Stage 3, decompression; Stage 4, post-dive.

Analysis of non-linear HRV indices, including SD1, SD1/SD2 ratio, and SampEn, revealed significant effects of dive stages after adjusting for age, pre-dive perceived stress, and respiratory rate (Table 5). SD1 and the SD1/SD2 ratio significantly increase across stages, with the most pronounced elevations at the bottom stage (*p* = 0.006 and 0.019, respectively).

**TABLE 5:** GEE analysis of group x time effects on non-linear HRV metrics across simulated diving stages in novice vs. experienced divers.

Variables	SD1				SD1/SD2				SampEn			
	*β*	95% CI		*p*	*β*	95% CI		*p*	*β*	95% CI		*p*
Age	−0.60	−1.21	0.01	0.053	−0.002	−0.01	0.004	0.574	0.002	−0.01	0.01	0.794
PSS	−0.19	−0.79	0.41	0.533	−0.005	−0.01	0.001	0.115	−0.01	−0.02	0.01	0.286
RR	−0.31	−1.24	0.62	0.513	0.02	0.01	0.02	<0.001	0.04	0.01	0.06	0.002
Diving stage				<0.001				<0.001				0.001
4 vs. 1	5.59	−3.33	14.51	0.220	0.05	−0.02	0.13	0.175	−0.04	−0.35	0.27	0.811
3 vs. 1	4.24	−1.88	10.36	0.174	0.04	−0.05	0.12	0.429	−0.004	−0.21	0.20	0.966
2 vs. 1	10.18	2.86	17.50	0.006	0.08	0.01	0.15	0.019	−0.08	−0.23	0.07	0.323
Experienced vs. novices	−3.32	−14.26	7.61	0.552	−0.06	−0.18	0.04	0.186	−0.19	−0.44	0.06	0.140
Experienced x diving stage				<0.001				<0.001				0.023
Experienced vs. novices at stage 4	10.12	−3.43	23.67	0.143	0.07	−0.04	0.17	0.199	0.23	−0.11	0.56	0.193
Experienced vs. novices at stage 3	12.30	1.81	22.79	0.022	0.17	0.06	0.27	0.002	0.36	0.11	0.61	0.005
Experienced vs. novices at stage 2	−8.62	−19.72	2.49	0.128	−0.003	−0.09	0.08	0.945	0.15	−0.10	0.40	0.236

Notes: SD1, Standard deviation 1 is the standard deviation of the distances of each data point from the line of identity (y = x) in the Poincaré plot; SD2, Standard Deviation 2 is standard deviation along the line of identity in the Poincaré plot; SD1/SD2 ratio, the ratio of short to long-term variability; SampEn, Sample entropy; RR, respiratory rate; Stage 1, pre-dive; Stage 2, bottom; Stage 3, decompression; Stage 4, post-dive.

Interactions between diver experience and diving stages significantly influenced non-linear HRV indices, including SD1, SD1/SD2 ratio, and SampEn (*p* < 0.001, *p* < 0.001, and *p* = 0.023, respectively; Figures 2F–H). These interactions were particularly significant during the decompression stage, where experienced divers demonstrated significantly greater increases in SD1 (*β* = 12.30, *p* = 0.022) and the SD1/SD2 ratio (*β* = 0.17, *p* = 0.001) than novices. Additionally, SampEn demonstrated the most significant group difference during decompression, with experienced divers maintaining higher HRV complexity than novices (*β* = 0.36, *p* = 0.005).

## Discussion

This study examined subjective and objective psychophysiological responses to deep diving in experienced and novice Navy divers. No significant differences were observed in self-reported anxiety and fear. However, significant interaction effects between diver experience and diving stages were found in objective measures, including salivary cortisol and amylase activity levels, and ANS regulation as reflected by linear and non-linear HRV indices. The most pronounced differences occurred during decompression, highlighting a critical period where experience may provide greater physiological stability. These results suggest that prior deep diving experience enhances psychophysiological adaptation and resilience to the demands of deep diving.

Deep diving poses significant challenges for Navy divers, primarily due to exposure to elevated ambient pressures and the demanding underwater environment. These conditions induce marked changes in psychophysiological indicators across various dive stages and during post-dive recovery (Colodro Plaza et al., 2014). The underwater setting has been recognized as a complex stimulus requiring robust psychological resilience to facilitate effective physiological and behavioral responses (Zec et al., 2023). In this context, psychological adaptability is increasingly acknowledged as critical to divers’ physiological regulation and mission performance. Excessive psychological stress or fear can compromise safety by prompting rapid ascents, potentially leading to decompression sickness or pulmonary barotrauma, thus jeopardizing operational outcomes (Morgan et al., 2004; Niewiedział et al., 2018). Diving to depths of 220 feet is especially challenging, particularly for individuals encountering such conditions for the first time. Prior research indicates that divers with greater experience demonstrate better emotional regulation and stress adaptation than their less experienced counterparts, underscoring the role of psychological preparedness in deep diving scenarios (Bonnet et al., 2008). In our study, participants undergoing simulated deep diving reported overall low levels of psychological distress, with a mean perceived stress score below 11 (out of 40), and very low scores on VAS for fear and anxiety. Although experienced divers reported slightly lower anxiety and fear levels than novices, these differences were not statistically significant. These findings are consistent with prior research involving recreational scuba divers, where participants self-reported exceptionally low psychological and physiological stress and perceived diving as a relaxing activity, despite exhibiting relatively high heart rates just prior to diving (Schaller et al., 2021). In the context of military diving, the minimal distress reported by our participants may be influenced by their professional responsibilities. Navy divers undergo rigorous training as part of their deployment readiness, and some may be hesitant to disclose feelings of fear or anxiety due to concerns that such disclosures could impact their performance evaluations, despite reassurances from researchers. Additionally, while all participants had general diving experience, those classified as novices may not have had prior exposure to deep diving, potentially influencing their psychological response patterns.

Self-reported psychological status can be influenced by individuals’ characteristics and personal coping resources (Smith, 2019). To complement these subjective measures, this study incorporated objective biomarkers, specifically salivary cortisol and amylase activity levels, to assess physiological stress responses to deep diving. Before diving, experienced deep divers exhibited significantly higher cortisol levels than novices. After adjusting for factors such as age and pre-dive perceived stress, significant interaction effects between diving experience and dive stage were identified. Specifically, experienced divers demonstrated more significant post-dive reductions in cortisol and amylase activity levels relative to novices. These findings align with previous research on stress response dynamics. Studies have shown that athletes often reach peak cortisol levels immediately before competition or while visualizing challenging scenarios (Bonifazi et al., 2000; Takai et al., 2004). Similarly, the stressors associated with deep diving may elicit emotional and cognitive responses that activate the hypothalamic-pituitary-adrenal (HPA) axis, resulting in elevated cortisol production (Zec et al., 2023). Following exposure to physical or psychological stressors, a subsequent decline in stress hormones such as cortisol and amylase reflects effective physiological adaptation and improved performance, which is more commonly observed in experienced individuals than novices (Meyer et al., 2015).

Although Navy dive tables are rigorously followed to ensure safe dive profiles through controlled descent and ascent protocols for managing nitrogen uptake and off-gassing, advanced underwater monitoring and a comprehensive understanding of divers’ physiological status to prevent diving-related disorders remain critical (Bube et al., 2022). HRV has been established as a reliable marker for monitoring ANS function and overall physiological status in divers (Schirato et al., 2020). Notably, persistently low HRV levels across diving stages have been linked to an elevated risk of known and undiagnosed cardiac conditions (Butler et al., 1993; Bai et al., 2013).

The ANS exhibits dynamic responses throughout the deep diving process. During the pre-dive stage, divers typically experience emotional and physiological activation of the ANS and hypothalamic-pituitary-adrenal (HPA) axis, characterized by increased heart rate and reduced HRV (Meyer et al., 2015; Bube et al., 2022). As descent progresses, the high-pressure environment elicits a more dominant parasympathetic response, driven by the trigeminocardiac reflex, baroreceptor activation, and cardiac stretch receptors (Panneton and Gan, 2020; Lundell and Ojanen, 2023). This response was evident in our findings, where global HRV indices increased significantly across all simulated diving stages, with the most pronounced changes observed at the bottom stage. Specifically, parasympathetic HRV indices were significantly elevated at depth (bottom stage), including linear measures (rMSSD, HF) and non-linear measures (SD1, SD1/SD2 ratio). These results are consistent with previous research under variable hyperoxia during open-circuit and closed-circuit diving, which reported increases in beat-to-beat intervals, SDNN, rMSSD, and HF at a depth of 30 m (Lafère et al., 2021). Additionally, significant variations in SampEn, a robust measure of HRV complexity, reflect the adaptability of the autonomic system to stressors (Richman and Moorman, 2000), were observed across diving stages in this study, suggesting that the physiological challenges of deep diving notably influence the complexity and dynamic regulation of the ANS.

Compared to novice deep divers and their pre-dive data, experienced divers consistently showed significantly lower mean heart rates and increased SDNN, rMSSD, LF, and HF across all diving stages. These results indicate enhanced parasympathetic modulation among experienced deep divers, corroborating previous research associating greater dive experience with increased parasympathetic activity (Lafère et al., 2021). Autonomic responses to high-pressure environments involve physiological adjustments and emotional stress-related reactions, potentially influencing the SNS activity (Lund et al., 2000). Experienced divers exhibited superior autonomic regulation during challenging stages, likely due to adaptive mechanisms developed from repeated exposures. This adaptive capacity suggests that targeted training and extensive diving experience foster physiological resilience and better equip divers to manage the psychophysiological demands associated with deep diving (Paganini et al., 2021). These findings underscore the critical role of experience in optimizing autonomic regulation during high-pressure underwater activities and underscore the need for further research and tailored training programs to enhance diver safety and performance. Interestingly, experienced divers demonstrated a marked increase in HRV indices during the post-dive phase, while novices exhibited relatively unchanged values. This suggests that experienced individuals may possess superior autonomic recovery mechanisms following the physiological and psychological demands of deep diving. Prior research indicates that repeated exposure to stressors can enhance vagal reactivation and improve stress-buffering capacity (Stanley et al., 2013; Lafère et al., 2021; Paganini et al., 2021). These post-dive changes may reflect trained autonomic plasticity, enabling more efficient rebalancing of the ANS following stress.

Following exposure to hyperbaric environments, decompression has been reported to induce various physiological alterations, including impaired endothelial function, immune activation, and inert gas bubble formation (Thom et al., 2015). These decompression-related changes are associated with increased levels of harmful microparticles, negatively correlating with pre- to post-dive changes in HRV measures such as SDNN and HF (Schirato et al., 2020). In this study, novice divers exhibited reduced autonomic adaptation across the diving stage compared to experienced divers, particularly during decompression, as reflected by more minor changes in linear and non-linear parasympathetic HRV indices. Additionally, a more significant reduction in cardiac complexity, represented by SampEn, was observed among novice divers during the Decompression stage. This finding suggests that decompression imposes more tremendous physiological strain on novice divers, leading to reduced autonomic adaptability characterized by lower HRV complexity (Lundell and Ojanen, 2023). These findings align with prior research demonstrating that diving-induced physiological stress reduces HRV complexity (Berry et al., 2017). Such outcomes underscore the critical importance of properly managing staged ascents to facilitate safe nitrogen elimination, as inadequate decompression may result in severe, life-threatening complications. Future research should prioritize developing real-time HRV monitoring systems to enhance diver safety and reduce decompression-related risks, particularly among novice divers (Schirato et al., 2020). Collectively, these findings emphasize the role of diving experience in enhancing physiological resilience, underscoring the need for tailored training programs and continuous monitoring strategies to optimize safety in naval diving operations.

### Limitations

This study’s data robustly supports the notion that deep diving experience can enhance psychophysiological adaptation and resilience, enabling divers to cope with high-pressure and stressful environments. However, several limitations need to be addressed in future research. Firstly, despite efforts to control factors such as the timing of sample collection, dietary intake, and oral hygiene practices, we could not fully account for all potential confounders affecting salivary cortisol and amylase levels, such as variations in sleep quality, recent physical or mental workload, and individual psychological traits. Future studies should incorporate comprehensive control or monitoring of these factors to enhance the reliability and interpretability of biomarker measurements. Second, although divers were instructed to remain still and minimize movement during HRV measurements, the recordings were conducted under non-resting conditions and may still have been affected by unavoidable movements during descent and ascent. These factors could introduce some variability in HRV readings, potentially influencing the interpretation of autonomic responses across diving stages. Future studies should consider incorporating motion sensors or synchronized movement tracking to better account for and control physical activity during underwater HRV data collection. Third, although the portable HRV device offered a non-invasive means to monitor autonomic activity during the dive, respiratory rate, an important confounder influencing HRV accuracy, was not directly measured due to equipment constraints. Instead, respiratory rate was estimated from beat-to-beat intervals using validated algorithms within Kubios software (Rogers et al., 2022). Despite the reliability of this estimation approach in controlled settings, direct measurement of respiratory rate during dives could enhance the precision of HRV analyses. Future studies should integrate real-time respiratory monitoring during underwater assessments to improve data accuracy. Fifth, cumulative diving exposure may compromise the ANS responses and performance (Berry et al., 2017); however, this study did not record the interval since participants’ last dive. Future studies should document and account for this variable to minimize potential confounding effects. Sixth, women represent a small proportion of Navy divers in Taiwan; consequently, only one female diver was included in this study. Therefore, the findings may not be generalizable to the broader population of female divers.

## Conclusion

This study reveals distinct physiological and regulatory differences between experienced and novice Navy deep divers. Experienced divers demonstrated more sophisticated autonomic and cardiovascular responses to deep diving stress, reflected in distinct HRV profiles and more significant post-dive reductions in stress biomarkers, specifically salivary cortisol and amylase activity. These findings highlight the importance of diving experience in building physiological resilience to high-pressure environments. Although no significant differences were found in self-reported psychological measures, this may reflect limitations in subjective reporting rather than a genuine absence of psychological stress. Future research should incorporate more sensitive or objective psychological evaluations. From a practical standpoint, training programs may benefit from incorporating strategies that support autonomic regulation and stress adaptation, such as progressive exposure, HRV-guided monitoring, and simulation-based training. Integration of real-time physiological monitoring may further improve diver safety and performance, especially for divers with limited experience in deep diving.

## Data Availability

The original contributions presented in the study are included in the article/supplementary material, further inquiries can be directed to the corresponding authors.
